# Latent tuberculosis infection and associated risk factors among undergraduate healthcare students in Italy: a cross-sectional study

**DOI:** 10.1186/1471-2334-13-443

**Published:** 2013-09-23

**Authors:** Paolo Durando, Giovanni Sotgiu, Fabio Spigno, Mauro Piccinini, Giovanni Mazzarello, Claudio Viscoli, Francesco Copello, Alessandro Poli, Filippo Ansaldi, Giancarlo Icardi

**Affiliations:** 1 Department of Health Sciences, Associate Professor of Hygiene, Preventive Medicine and Public Health, Hygiene Unit, IRCCS AOU San Martino-IST teaching Hospital, University of Genoa, Via Antonio Pastore 1, 16132, Genoa, Italy; 2 Department of Biomedical Sciences, Associate Professor of Medical Statistics, Research, Medical Education and Professional Development Unit, AOU Sassari, University of Sassari, Via Padre Manzella 4, 07100, Sassari, Italy; 3 Department of Health Sciences, Associate Professor of Occupational Health, Chief of the Occupational Health Unit, IRCCS AOU San Martino-IST teaching Hospital, University of Genoa, Largo Rosanna Benzi 10, 16132, Genoa, Italy; 4 Prevention and Protection Service of the University of Genoa, Via Balbi 5, 16126, Genoa, Italy; 5 Department of Health Sciences, Infectious Diseases Unit, IRCCS AOU San Martino-IST teaching Hospital, University of Genoa, Largo Rosanna Benzi 10, 16132, Genoa, Italy; 6 Department of Health Sciences, Full Professor of Infectious Diseases, Chief of the Infectious Diseases Unit, IRCCS AOU San Martino-IST teaching Hospital, University of Genoa, Largo Rosanna Benzi 10, 16132, Genoa, Italy; 7 Occupational and Preventive Medicine Unit, IRCCS AOU San Martino-IST teaching Hospital, Largo Rosanna Benzi 10, 16132, Genoa, Italy; 8 Department of Health Sciences, Full Professor of Hygiene, Preventive Medicine and Public Health, Chief of the Hygiene Unit, IRCCS AOU San Martino-IST teaching Hospital, University of Genoa, Via Antonio Pastore 1, 16132, Genoa, Italy

**Keywords:** Tuberculosis, Latent tuberculosis infection, Tuberculin skin test, Interferon-gamma release assay, Healthcare students, Risk-factors

## Abstract

**Background:**

The screening of both healthcare workers and students attending teaching hospitals for latent tuberculosis infection (LTBI) is recommended in hospitals of many countries with a low-incidence of TB, including Italy, as a fundamental tool of tuberculosis (TB) control programs. The aim of the study was to estimate the prevalence of LTBI and evaluate the main risk-factors associated with this condition in a cohort of healthcare Italian students.

**Methods:**

In a cross-sectional study, performed between January and May 2012, 881 undergraduate students attending the Medical, Nursing, Pediatric Nursing and Midwifery Schools of the University of Genoa, trained at the IRCCS San Martino-IST Teaching Hospital of Genoa, were actively called to undergo the Tuberculin Skin Test (TST). All the TST positive cases were also tested with an Interferon-Gamma Release Assay (IGRA) to confirm the diagnosis of LTBI. A standardized questionnaire was collected for risk-assessment analysis.

**Results:**

Seven hundred and thirty-three (83.2%) subjects underwent TST testing. The prevalence of TST positives was 1.4%, and in 4 (0.5%) out of 10 TST positive cases LTBI diagnosis was confirmed by IGRA. No difference in the prevalence of subjects who tested positive to TST emerged between pre-clinical (n = 138) and clinical (n = 595) students. No statistically significant association between TST positivity and age, gender, and BCG vaccination was observed. The main independent variable associated with TST positivity was to be born in a country with a high TB incidence (i.e., ≥20 cases per 100,000 population) (adjusted OR 102.80, 95% CI 18.09-584.04, p < 0.001).

**Conclusions:**

The prevalence of LTBI among healthcare students resulted very low. The only significant association between TST positivity and potential risk factors was to be born in high TB incidence areas. In countries with a low incidence of TB, the screening programs of healthcare students before clinical training can be useful for the early identification and treatment of the sporadic cases of LTBI.

## Background

Latent tuberculosis infection (LTBI) and active tuberculosis (TB) represent occupational risks among healthcare workers (HCWs), even in low TB incidence areas, such as the European Union/European Economic Areas, where notification rates of 14.6 per 100,000 population were reported in 2010, and several Member States showed figures lower than 10 per 100,000 population [1-4]. It has been proven that the majority of work-related active cases occur when the infection risk is not suspected and preventive measures are not taken in the healthcare sector [5,6]. Moreover, the risk of TB transmission by HCWs to patients also exists, as recently documented in our country by a nosocomial case from a nurse to a patient occurred in a large Teaching Hospital [7]. The lack of knowledge about TB transmission, preventive and biosafety measure, and diagnosis of infection and disease has been reported among professionals and students of healthcare settings [8].

Healthcare students involved in clinical training could be exposed to occupational risks similar to those of HCWs. Therefore, the screening for LTBI of both healthcare workers and undergraduate students attending teaching hospitals is recommended especially in low-incidence countries, including Italy, in order to obtain an early diagnosis of cases and prevent progression to active disease [9,10]. Despite the higher risk of TB infection reported in high TB burden countries among both HCWs and healthcare students compared to the general population [3,11], such TB control activities are difficult to be implemented in these settings [12].

Very few studies have investigated the epidemiology of TB infection among undergraduate healthcare students worldwide, and none, with a large sample, in areas with a low-incidence of TB [11,13-17].

In the quest to bridge this gap, a survey to assess the prevalence of the individuals with a LTBI and measure the main risk-factors associated with TST positivity was performed on undergraduate students, either attending or not the hospital wards, trained at a large teaching hospital in Northern Italy.

## Methods

### Study design and setting

The survey was carried out, between January and May 2012, at the IRCCS AOU San Martino-IST Teaching Hospital of Genoa, Italy, the regional tertiary adult acute care reference hospital with a 1,400 bed capacity. Almost all the cases of infectious TB that occur in the Liguria Region, where Genoa is located, are hospitalized at the Infectious Diseases Unit of the IRCCS San Martino-IST Teaching Hospital, since it is the only facility with negative-pressure rooms for contagious patients.

All students attending the last 3 years of the Medical School (clinical students) and all students attending the first year of Nursing, Pediatric Nursing and Midwifery Schools (pre-clinical students) were actively summoned to undergo LTBI testing. Clinical students attended different departments of the hospital, including the infectious disease wards.

### Diagnostic methods, management of TST positive cases and questionnaire items

Tuberculin Skin Test (TST) was performed by trained HCWs using the *Mantoux* technique. Plastic disposable syringes and short bevel needles (25-gauge) were used to intradermally inject 0.1 mL of purified protein derivative (PPD-Rt 23, 2 Tubercoline Units, Staten Serum Institut, Copenaghen, Denmark) into the volar surface of the middle third of the forearm of each subject. The skin was slightly stretched, and the needle held almost parallel to the skin surface with the bevel upwards. A dose of 0.1 mL was slowly injected into the superficial layer of the dermis and a small, blanched papule with a diameter of 8 – 10 mm in diameter appeared, disappearing after approximately ten minutes. The maximum diameter of palpable induration was measured after 48–72 hours: a positive TST was defined as an induration measuring ≥ 10 mm in healthy subjects.

All the TST positive cases were also tested with an Interferon-Gamma Release Assay (IGRA; QuantiFERON® TB-Gold Cellestis, Carnegie, Australia) to confirm the diagnosis of LTBI, because of its major specificity compared with conventional TST [18], as also recommended by other countries [19,20].

All the IGRA positive cases were carefully examined by an infectious diseases specialist and underwent chest radiography. Furthermore, they were counseled on clinical signs and symptoms of active TB, and strongly recommended to immediately report their onset to the Hygiene and Occupational Health Units of the IRCCS AOU San Martino-IST Teaching Hospital.

Information about age, gender, nationality, health status, years of attendance in hospital wards, and previous exposure to *Bacille Calmette-Guérin* (BCG) vaccine was obtained using a standardized questionnaire.

### Ethical issues

All the activities were performed in compliance with the Declaration of Helsinki and current healthcare standards according to the recommendations of the Italian Ministry of Health [10]. All students included in the survey were informed by a physician about the rationale and aims of the survey, and a written informed consent was obtained. According to Italian legislation concerning the guidelines on observational studies, ethical approval for conducting this survey was unnecessary, and on this basis, cross-sectional studies do not require a formal approval by local Institutional Review Boards [21]. However, the study was regularly notified to the Ethic Committee of the IRCCS AOU San Martino-IST Teaching Hospital of Genoa, Italy. Personal information regarding the subjects included in the study was protected according to Italian law [22]. The study was part of the “2012 Risk Assessment Management Program” of the IRCCS AOU San Martino-IST Teaching Hospital.

### Statistical analysis

All the covariates mentioned in the previous description of the study were collected in an *ad-hoc* e-form.

The distribution of the qualitative variables was shown in percentages, whereas the quantitative variable means and standard deviations were used after performing the Shapiro-Wilk test and confirmed their parametric distribution. Categorical variables were compared using the Chi-squared test. Logistic regression analysis was performed to assess the association between LTBI and potential covariates. Results were stratified according to gender, nationality, TB incidence of the country of birth, clinical years of attendance, and BCG vaccination for the risk-assessment analysis. P-values ≤0.05 were considered statistically significant.

All the analyses were carried out using Stata statistical software (StataCorp, Stata Statistical Software Release 9, College Station, TX, USA, 2005).

## Results

From January to May 2012, 881 healthcare students attending the Medical, Nursing, Pediatric Nursing and Midwifery Schools were selected for the survey. Of these, 733 (83.2%) students performed TST screening according to the procedures described above, while 148 (16.8%) students did not answer the summons. The main demographic and epidemiological characteristics of the study population are shown in Table 1[23].

**Table 1:** Demographic, epidemiological and clinical characteristics of a cohort of healthcare students trained at a regional tertiary adult acute care reference hospital in Italy

**Variables**		**n = 733**
Mean (SD) age, year		23.6 (3.1)
Males, n (%)		279/733 (38.1)
Country of birth, n (%)	Italy	692/733 (94.4)
	Israel	12/733 (1.6)
	Albania	9/733 (1.2)
	Others	20/733 (2.7)
Born in a high TB incidence country*, n (%)		12/733 (1.6)
Potential professional exposure, n (%)	Clinical students	595/733 (81.2)
	Preclinical students	138/733 (18.8)
Year of attendance, n (%)	Fourth	270/733 (36.8)
	Fifth	169/733 (23.1)
	Others	294/733 (40.1)
BCG immunization, n (%)		13/733 (1.8)
Positive TST, n (%)		10/733 (1.4)
Positive IGRA, n (%)		4/10 (40)

*SD* Standard Deviation.

*BCG* Bacille Calmette-Guérin.

*TST* Tuberculin Skin Testing.

*IGRA* Interferon-γ Release Assay.

*High Incidence: ≥20 cases per 100,000 population (according to reference 23).

The majority of the healthcare students were females (454/733, 61.9%) and born in Italy (692/733, 94.4%), with a mean (SD) age of 23.6 (3.1) years. Only 12 (1.6%) subjects enrolled in the survey were born in a country characterized by a high TB incidence (i.e., ≥20 cases per 100,000 inhabitants yearly). BCG immunization had been previously performed in 13 (1.8%) out of the 733 students: 5 students came from countries at a high TB incidence. The proportion of positivity to TST was 1.4% (10/733). Four (0.5%) out of 10 TST positive students had an IGRA positive result. More than 80% of the students enrolled in the survey were exposed to patients during their clinical training activities, but none of the cases of LTBI reported a previous professional or household contact with a confirmed case of infectious TB.

TST positivity proportion resulted significantly higher in migrants (9.8% VS. 0.9%; p-value = 0.001) and in those born in high incidence countries (33.3% VS. 0.8%; p-value <0.0001) (Table 2) [23].

**Table 2:** Proportion of positive tuberculin skin testing response stratified by the main collected variables

**Variables**		**TST**	**p-value**
		**n (%), 95% CI**	
Gender	Male	3/279 (1.1), -0.001 – 0.023	0.75
	Female	7/454 (1.5), 0.004 – 0.026	
Nationality	Italian	6/692 (0.9), 0.002 – 0.016	0.001
	Migrant	4/41 (9.8), 0.007 – 0.189	
TB incidence of the country of birth*	≥20 cases per 100,000 population	4/12 (33.3), 0.066 – 0.600	<0.0001
	<20 cases per 100,000 population	6/721 (0.8), 0.002 – 0.015	
Potential professional exposure calculated without the interference of subjects coming from countries at high-incidence of TB	Pre-clinical students	3/136 (2.2), -0.003 – 0.046	0.09
	Clinical students	3/585 (0.5), -0.001 – 0.011	
BCG immunization	Not vaccinated	9/720 (1.3), 0.005 – 0.021	0.17
	Vaccinated	1/13 (7.7), -0.068 – 0.222	

*CI* Confidence Intervals.

*BCG* Bacille Calmette-Guérin.

*TST* Tuberculin Skin Testing.

*High Incidence: ≥20 cases per 100,000 population (according to reference 23).

The logistic regression analysis confirmed the increased probability of a positive TST response in migrants (adjusted OR 16.05, 95% CI 3.63-70.91, p-value <0.001) and in students born in high TB incidence countries (adjusted OR 102.80, 95% CI 18.09-584.04, p-value <0.001) (Table 3) [23].

**Table 3:** Association between a positive tuberculin skin testing response and potential independent variables

**Immunological assay**	**Univariate analysis**		**Adjusted analysis**	
	**OR**	**p-value (95% CI)**	**OR**	**p-value (95% CI)**
**Tuberculin skin testing**				
Increasing age, year	1.09	0.10 (0.98–1.21)	1.10	0.09 (0.99–1.22)
Male	0.69	0.60 (0.18–2.71)	0.66	0.56 (0.17–2.61)
Migrant	12.36	<0.001 (3.34–45.7)	16.05	<0.001 (3.63–70.91)
Born in a high TB incidence country*	59.58	<0.001 (14.06–252.58)	102.80	<0.001 (18.09–584.04)
BCG immunization	6.58	0.09 (0.77–56.14)	6.72	0.08 (0.78–57.69)

*OR* Odds Ratio.

*CI* Confidence Intervals.

*BCG* Bacille Calmette-Guérin.

*High Incidence: ≥20 cases per 100,000 population (according to reference 23).

Chest radiography was performed in all individuals with a positive IGRA response and resulted negative.

## Discussion

The transmission of *Mycobacterium tuberculosis* in healthcare settings to both HCWs and patients is a well-documented threatening event, and is most likely to occur from unrecognized or inappropriately treated TB cases [2,3]. Programs for the screening and treatment of LTBI cases within HCWs, combined with other interventions aimed at reducing the risk of nosocomial transmission, represent fundamental tools of TB control programs and are strongly recommended in many countries, including Italy [8,9], where an annual TB incidence of 4.9 per 100,000 population was estimated in 2010 [23].

A recent systematic review reported that the median estimated annual risk of LTBI among HCWs was 2.9% in low-incidence countries, against an estimated risk of 7.2% in countries with a high TB incidence [3].

Despite healthcare students involved in clinical training may run risks of being exposed to *Mycobacterium tuberculosis* similar to HCWs within the hospital setting, very few studies have addressed this issue. To the best of our knowledge, this is the first study that investigated, using a large sample, both the prevalence of LTBI and the main risk factors associated with TST positivity in a cohort of students, either attending or not the hospital wards, trained at a large teaching hospital located in a low TB incidence area.

Studies performed in high incidence countries have reported LTBI prevalence figures ranging from 9.2% to 72% among healthcare students [11,14,16,17]: TST was used for the diagnosis of LTBI in these surveys.

Our sample was characterized by a very low prevalence of TST positive cases among students (1.4%). The diagnosis of LTBI was confirmed by IGRA testing (0.5%), thus reducing the potential occurrence of “false positive” cases due to exposure to atypical mycobacteria or BCG vaccination. In a German study, performed between 2005 and 2009, none of the 110 trainees or young healthcare professionals screened with an IGRA testing showed positive results [24]. This result, obtained in a country with a low incidence of TB, clearly confirms our findings.

No difference with respect to the prevalence of TST positivity emerged when comparing pre-clinical with clinical students: the data show a very low prevalence of LTBI among students during clinical training, in accordance with the data observed among healthcare students at the beginning of the training period in various occupations (nurses, medical students, physical therapists) by other authors in Europe [25]. Our findings greatly diverged from the results obtained in high-to-intermediate TB incidence areas, which reported an increased risk of *Mycobacterium tuberculosis* infection in senior medical and nursing students ranging between 2.2 and 3.8 [14,16].

The low incidence of TB in our hospital (11 cases of infectious TB patients hospitalized in 2012), the usual precautions taken to avoid the exposure of students to known infectious TB cases during the training activities, and recommendations on the proper use of individual protection devices and measures at our hospital may explain the observed results.

BCG immunization was rarely recorded in the study sample, consistently with the current Italian guidelines for TB prevention that recommend vaccination of HCWs and students only in selected cases, based on risk-assessment at hospital level (i.e., individuals unavoidable exposure to highly contagious multidrug-resistant TB cases and individuals with contraindications to LTBI preventive treatment) [10]. BCG immunization was not associated with a positive TST result: this lack of association has previously been reported also by other authors [17].

The risk assessment analysis clearly demonstrated that coming from a geographical area with a high TB incidence was actually a major risk factor for TST positivity among healthcare students (adjusted OR = 102.80). An association between foreign birth and LTBI has also been observed by other authors in Europe [24,26]. The migration of students from low- and middle-income countries to high-income countries is part of the relatively recent globalization phenomenon that is expected to increase in the near future. Our findings highlight the need to design and implement effective TB infection control programs specifically for these students in the healthcare facilities of Western countries [18].

### Limits

The main limit of our survey was the cross-sectional study design. For this reason, changes over time could not be monitored. Moreover, a single-step TST procedure was used, although IGRA testing was systematically carried out in the event of TST positivity, thus increasing the specificity of the confirmed diagnosis of LTBI. Another limit involved the difficulty to obtain adequate information concerning the time spent by the students in the hospital before being tested and their specific exposure to confirmed cases of infectious TB, both at professional and at community level (family, social activities, etc.). Additionally, a lack of demographic and epidemiological information concerning the students who refused to enter the survey existed. A further selection bias of the study population with respect to the attendance of hospital wards, between medical (clinical) and nursing/midwifery (pre-clinical) students, prevented any specific risk assessment for TB infection in the different healthcare schools.

## Conclusions

In summary, the prevalence of LTBI among undergraduate Italian healthcare students was very low and the only significant association between TST positivity and potential risk factors was to be born in high TB incidence areas. In countries with a low incidence of TB, our results confirm that LTBI screening programs should include healthcare students before clinical training for early identification and treatment of the sporadic cases of LTBI.

## Abbreviations

TB: Tuberculosis; LTBI: Latent tuberculosis infection; TST: Tuberculin skin test; IGRA: Interferon gamma release assay; BCG: Bacille calmette-guèrin; HCWs: Healthcare workers; PPD: Purified protein derivative.

## Competing interests

The authors declare that they have no competing interests.

## Authors’ contributions

PD, FS, CV, FC and GI made substantial contributions to the conception and design of the study and were involved in writing and drafting the manuscript. GS, AP and FA made substantial contributions to the analysis and interpretation of data and were involved in revising the manuscript critically for important intellectual content. They were involved in drafting the manuscript. FS, MP and GM were involved in performing the clinical activities as well as in the analysis and interpretation of data. They were involved in drafting the manuscript. All authors read and approved the final manuscript.

## Pre-publication history

The pre-publication history for this paper can be accessed here:

http://www.biomedcentral.com/1471-2334/13/443/prepub
